# Re-imagining sensory substitution through gestural control: Point-To-Tell 2

**DOI:** 10.1080/17483107.2025.2590679

**Published:** 2025-11-20

**Authors:** Ligao Ruan, Giles Hamilton-Fletcher, Mahya Beheshti, Todd E. Hudson, Maurizio Porfiri, John-Ross Rizzo

**Affiliations:** a Department of Mechanical and Aerospace Engineering, NYU Tandon School of Engineering, Brooklyn, NY, USA; b Department of Rehabilitation Medicine, NYU Grossman School of Medicine, New York, NY, USA; c Department of Radiology, NYU Grossman School of Medicine, New York, NY, USA; d Center for Urban Science and Progress, NYU Tandon School of Engineering, Brooklyn, NY, USA; e Department of Biomedical Engineering, NYU Tandon School of Engineering, Brooklyn, NY, USA

**Keywords:** Assistive technology, object detection, human–computer interaction, sensory substitution, visual impairment

## Abstract

**Aim:**

Sensory substitution devices (SSDs) can convert environmental information into an accessible format for people who are blind or have low vision (pBLV). Yet, current SSDs often passively deliver all of the information available with limited user control, potentially leading to confusion and/or cognitive overload. To address this issue, this work proposes a selective, gesture-controlled system intended to improve information relevance and reduce cognitive overload.

**Methods:**

We present Point-To-Tell 2, a system that enables pBLV to privately and efficiently select which information to convey through simple pointing-based gestural control. By integrating a monocular camera with AI-driven pipelines for depth estimation, hand pose tracking, and object detection/segmentation, the system identifies the users’ 3D pointing direction and announces the names and distances of objects as they are pointed at, thereby connecting an object’s spatial position and identity through hand proprioception.

**Results:**

Validation tests in controlled indoor environments show high hand pose tracking accuracy, ensuring reliable ray-casting and object selection despite declining object recognition at longer distances. Distance estimates are stable at close range, though a systematic bias is present.

**Conclusion:**

This work introduces and technically validates an assistive system designed to improve the usability of assistive technologies by focusing system feedback—potentially reducing users’ cognitive load and enhancing their spatial comprehension by leveraging concurrent hand proprioception. Future work will involve user testing and expanding system features to further enhance its practicality across more diverse scenarios.

## Introduction

Sensory substitution devices (SSDs) can convert visuospatial information from the environment into patterns of auditory [1,2] or tactile stimulation [3,4]. This process makes visual information more accessible for people who are blind or have low vision (pBLV), as they receive information through alternative modalities to vision, such as hearing or touch. While these technologies can partially support tasks like object localisation, navigation, and environmental awareness [5–7], they often passively provide continuous or generic feedback that does not adapt to users’ immediate goals or intentions. As a result, there is an abundance of irrelevant background information that is not explicitly tied to the user’s existing spatial reference frame, and not conducive for pBLV to form robust mental maps of their surroundings [5]. To address these limitations, more adaptive and user-driven solutions for sensory substitution are needed, tailored to specific tasks, contexts, and user needs.

Decades of research in sensory substitution have generated a wide array of assistive technologies aimed at improving the lives of pBLV [8–10]. In recent years, breakthroughs in artificial intelligence (AI), particularly with deep learning algorithms in computer vision, have made it possible to interpret visual images in a more meaningful manner to the user (e.g., text, object categories, and distances) before translating this information into alternative modalities [11–15]. Among these solutions, some have leveraged finger tracking so users can designate specific targets within the image [16–18], thereby offering a more focused form of sensory substitution (e.g., reading text at the user’s fingertip location). However, such systems often lack robust object recognition capabilities, operate effectively only within peripersonal space, or require the user to explicitly consider and adjust the camera viewpoint. These constraints underscore the need for more adaptive, intuitive, and user-driven approaches that deliver richer, more context-aware feedback, ultimately pushing sensory substitution to become a more seamless extension of everyday life.

An ideal version of sensory substitution would operate as an extension of the user’s natural three-dimensional movements and inquiries about the environment, providing relevant and appropriate supplementary information while also being non-obstructive during daily manual tasks such as shopping, cooking, and housekeeping. Khambadkar et al. [17] designed a system that employs a wearable Microsoft Kinect infra-red depth-sensing camera that hangs from the user’s neck and rests on the sternum. The system analyzes the RGB-D image, which provides colour classification, distances, and uses AI to recognise both humans in the image as well as different hand gestures from the user. The user selects which information to convey (colour, humans, distance) through different hand gestures and the system communicates this information near the user’s two-dimensional fingertip location in the image. However, the system lacks object identification capabilities, is implemented on a desktop computer, and provides false labels during angled finger orientations. The FingerReader system [18], introduced by Shilkrot et al. is a finger-worn device equipped with a small camera to assist users in reading printed text. By scanning lines of text with their finger, users receive real-time auditory and tactile feedback, enabling efficient text access and document navigation. While this innovative design highlights the potential of wearable technology for enhancing user interaction, it faces challenges related to user comfort during prolonged use, the high level of precision required for effective text scanning, and the need for further refinement in robustness and usability across diverse real-world conditions. Chen et al. [19] introduced a wearable device disguised as a neckband headphone, designed to assist pBLV users in locating and reaching surrounding objects. It integrates a binocular camera for 3D depth sensing, speech guidance for user interaction, and object recognition algorithms for identifying and tracking target objects like cups, keys, or phones. Although preliminary tests demonstrated its effectiveness for close-range scenarios, the evaluation focused on a limited range of tasks and distances. Consequently, while the device showed promise, its broader usability and performance in more varied or extended situations remain unverified.

To further advance sensory substitution, we present a new combinatorial AI framework building upon our previous prototype [20] that harnesses depth estimation [21], hand pose estimation [22], and object segmentation [23]. This is used to go beyond previous approaches (that use the fingertip’s X and Y position in the frame) to instead simulate the actual pointing direction of the user in 3D space, creating a ‘virtual laser pointer’ that extends from the user’s pointing finger, which is then used to select key information in the environment. This is done with a view to both improving the efficiency of acquiring information from the environment while also helping to integrate this information into the user’s own pre-existing sense of space, ultimately with the goal of improving spatial awareness for pBLV. This gesture-based approach refines how spatial details can be delivered, with the goal of supporting more efficient mental mapping of the environment for pBLV and ultimately moving sensory substitution towards a more user-driven, practical solution.

The key contributions of this work are as follows:

1. We introduce a gesture-focused sensory substitution framework that handles three-dimensional gesture analysis (combining depth and hand pose estimation) and object recognition from a single monocular camera mounted on the user’s backpack shoulder strap. By combining both processes in one feed, our system delivers a user-centric solution that enhances on-demand spatial recognition for pBLV.
2. Rather than scanning the entire image frame, the system focuses detection and analysis only on the region indicated by the three-dimensional pointing gesture. This adaptive Region-of-Interest (ROI) strategy reduces extraneous computation, minimises the potential for sensory overload, and offers more natural, proprioceptively guided feedback. As a result, pBLV receive precise information tied directly to their immediate area of interest.
3. We conduct validation tests to evaluate the accuracy of our gesture-focused sensory substitution system at various distances (1m to 5m) and horizontal offsets (±1.5 m). Our hypothesis was that the system would achieve high target recognition accuracy at all test angles and depths, validating performance in three-dimensions and ultimately improving the resolution of the sensory substitution.

## Methods

### Participant

A validation test was conducted with one 25-year-old male participant who had normal vision. Prior to the experiment, the participant was trained on the system to eliminate any potential bias stemming from unfamiliarity. While the participant’s vision was normal, the experimental design aimed to evaluate the technical accuracy of the system, specifically in its ability to detect and recognise objects based on pointing gestures, without being influenced by user-specific factors or variation.

### Equipment

The system is engineered as a portable assistive technology solution tailored for pBLV. The hardware configuration includes an IMX291 2MP USB monocular camera, attached to the front shoulder strap of a standard backpack, providing a front-facing 160° diagonal field of view to capture the user’s surroundings. Computational tasks are performed on an NVIDIA Jetson Orin NX, securely housed within the backpack for portability. The system is powered by a Krisdonia 25,000mAh power bank, ensuring uninterrupted operation during extended use. Audio feedback is delivered through SHOKZ OpenComm2 Open-Ear Bone Conduction Headphones, enabling users to receive object information without compromising environmental awareness [24–27].

### Combinatorial AI framework

**Figure 1.**
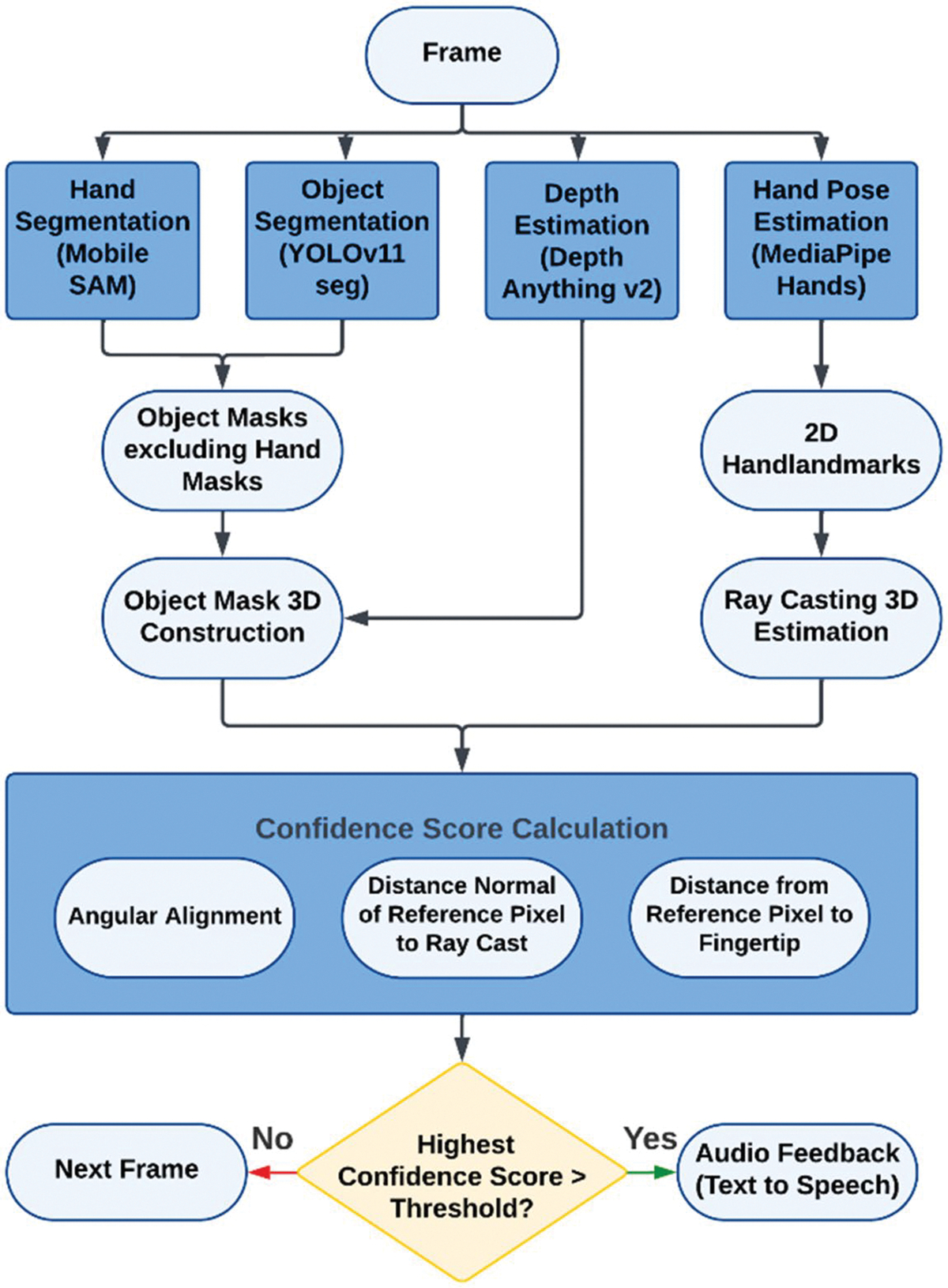
The system’s multimodal framework for determining the object pointed at by the user. The pipeline begins with frame acquisition, followed by parallel processing modules: Hand Segmentation (MobileSAM) for isolating hands, Object Segmentation (YOLOv11) for detecting objects, Depth Estimation (Depth Anything v2) for spatial representation, and Hand Pose Estimation (MediaPipe Hands) for extracting 2D hand landmarks. Outputs are integrated to construct 3D object masks and estimate the pointing ray. A confidence score is calculated based on angular alignment, distance normal of reference pixel to ray cast, and distance from reference pixel to fingertip. The object with the highest confidence exceeding the threshold is identified, triggering audio feedback, while frames without qualified objects proceed to the next iteration.

This section introduces a combinatorial AI framework designed to identify objects selected through finger-pointing by the user. The system analyzes the user’s hand pose and finger orientation to determine the three-dimensional pointing direction while simultaneously detecting and segmenting objects. The pipeline begins with frame acquisition, followed by parallel modules for hand segmentation (MobileSAM), object segmentation (YOLOv11), depth estimation (Depth Anything v2), and hand pose estimation (MediaPipe Hands). Outputs are integrated to construct 3D object masks and estimate the pointing ray. A confidence score, based on angular alignment, distance normal of reference pixel to ray cast, and distance from reference pixel to fingertip, identifies the intended object, triggering audio feedback if the score exceeds a threshold. This framework involves both sequential and parallel processes, as illustrated in Figure 1.

### Hand-pose estimation

Hand pose estimation serves as a critical component of the system, enabling detection and interpretation of the user’s hand pose by estimating the 3D position of multiple hand landmarks. This process leverages MediaPipe Hands, a state-of-the-art framework for hand landmark detection [28]. MediaPipe Hands model identifies 21 key landmarks on each hand, including critical joints such as the Distal Interphalangeal Joint (DIP) and Proximal Interphalangeal Joint (PIP) and Metacarpophalangeal Joints (MCP) of the index finger. These landmarks are then used to estimate the pointing direction in 3D space.

The MediaPipe Hands model employs a two-stage architecture designed for efficiency and accuracy in real-time applications. The first stage is a single-shot palm detector, which uses a convolutional neural network (CNN) [29] based on the Single Shot Multibox Detector framework [30], localising the palm’s bounding box in the frame. This detection step is lightweight and optimised to quickly identify potential hand regions. The second stage utilises a regression-based neural network to predict 21 hand landmarks in 3D, including the wrist, knuckles, and fingertips, from the cropped hand region identified in the first stage. This landmark model employs depthwise separable convolutions to reduce computational cost while maintaining high accuracy. By combining these stages, the architecture ensures efficient, robust, and real-time hand pose estimation, capable of handling varying hand gestures and lighting conditions.

To compute the pointing direction, the MCP-to-DIP line of the index finger is extracted, forming the basis for ray-casting calculations. The detected hand poses are processed frame by frame, ensuring that even dynamic and continuous gestures are captured accurately.

### Object and hand segmentation

The object segmentation module leverages the YOLOv11 instance segmentation model to analyse each video frame and detect objects within the scene [31]. YOLOv11, an advanced iteration of the YOLO family [32], has object detection and instance segmentation capabilities. For each detected object, the YOLOv11-seg model generates a bounding box, class label, and a segmentation mask. Unlike the bounding box, which provides a coarse localisation, the segmentation mask ensures pixel-level accuracy in object delineation. This capability is crucial for refining the spatial representation of objects, especially in complex scenes with overlapping or closely positioned objects. In our system, we integrate the YOLOv11 medium-sized pre-trained model which can predict 80 common object classes from the COCO dataset [33].

When the user points at an object, parts of their hand may overlap the object in the camera frame, leading to incorrect segmentation where some hand pixels are misclassified as belonging to the object by the YOLOv11 model. This overlap can affect the system’s accuracy, as it compromises the accuracy of 3D reconstruction in subsequent processing, potentially leading to erroneous object identification or misaligned spatial information. To address this issue, we incorporate MobileSAM [34], a lightweight and efficient variant of the Segment Anything Model (SAM) [35], designed to perform precise image segmentation tasks with a focus on portability and computational efficiency. Like the original SAM, MobileSAM supports prompts in the form of points or bounding boxes to guide segmentation. In our implementation, we utilise the index finger DIP and PIP detected by the hand pose estimation module as point prompts to segment the entire hand region.

Once the hand region is segmented by MobileSAM, we remove these pixels from the object segmentation masks predicted by YOLOv11. By filtering out the hand pixels, the segmentation results are refined to represent only the object, eliminating any interference caused by overlapping hand pixels. The removed hand pixels are discarded and not reassigned to any object class. This process enhances the system’s reliability and precision in spatial mapping, ensuring consistent performance in scenarios involving hand-object overlap.

### Depth estimation

Depth estimation is a pivotal step in the system, enabling the reconstruction of spatial information from a single monocular camera. Our system employs Depth Anything v2 [36] metric depth model, a deep learning-based model that generates depth maps from video frames. This process assigns depth values to every pixel in the input frame in metres, providing 3D spatial context for detected objects.

Depth Anything v2 is an advanced monocular depth estimation model that is trained exclusively on high-quality synthetic datasets generated by graphics engines. This approach enables the model to capture fine-grained details and handle challenging surfaces like transparency or reflectivity. For metric depth estimation, the pre-trained Depth Anything v2 encoder is fine-tuned with a simple Dense Prediction Transformer [37] head using synthetic datasets such as Hypersim [38] for indoor scenes, and Virtual KITTI [39], for outdoor environments. In the current system, we deployed the indoor metric depth small size model for depth estimation of detected objects.

The 3D space reconstruction process converts the 2D coordinates of detected object masks into 3D space by leveraging the depth map created by Depth Anything v2. Once an object is detected by the system, the distance will be determined by the pixel that represents that object. For each pixel in the mask generated by the object segmentation model, the depth value is used alongside the camera’s intrinsic parameters (focal lengths $f_{x}$, and $f_{y}$, and optical centre offsets $c_{x}$ and $c_{y}$) to compute real-world 3D coordinates $\left(X_{obj},Y_{obj},Z_{obj}\right)$ using Equation (1):

(1) $$X_{obj}=\frac{\left(x_{obj}\;-\;c_{x}\right)\cdot D}{f_{x}},Y_{obj}=\frac{\left(y_{obj}\;-\;c_{y}\right)\cdot D}{f_{y}},Z_{obj}=D$$

Where the $x_{obj},\;y_{obj}$ represent the frame coordinates of the pixel, D represents the depth of that pixel by depth estimation module.

During development, we noticed suboptimal performance when using the pretrained Depth Anything v2 metric depth model to accurately predict the depth of the user’s hands. As a result, we implemented a method to manually assign depth values to hand joints based on their 2D positions and dynamically calculated depth-scaling factors. This approach enables the reconstruction of pseudo-3D coordinates for the hand joints, which are essential for computing the 3D ray-casting direction used in object localisation. First, we obtain the pixel distances between key finger joints in the 2D image plane, specifically the distances from the wrist to the MCP joints, from the MCPs to the PIP joints, and from the PIP to the DIP joints. These distances are averaged, $d_{\mathrm{avg}}$, with Equation (2), providing a baseline gauge of how extended the finger is. We then map the depth of the finger within a predefined range, between a minimum depth $d_{\min}=0.2\;m$ and a maximum depth $d_{\max}=0.8\;m$, yielding a range constraint of the pointing depth. We refine this estimate by comparing the MCP-PIP segment, $d_{\mathrm{mcpip}}$, against $d_{\mathrm{avg}}$, as an indicator of finger orientation leveraging the fact that the wrist-MCP segment changes less than MCP -PIP when the pointing direction shifts in the frame. When the $d_{\mathrm{mcpip}}$ is relatively shorter compared to the $d_{\mathrm{avg}}$, it suggests the finger is extended in a manner that is more forward-facing and so we simulate a deeper depth for the ray-cast. When this segment is relatively longer, it indicates the finger is pointing sideways, so we simulate a shallower depth for the ray-cast. Here, we use the Depth Scaling to represent this relationship which is used to normalise how much the depth shifts based on finger configuration with Equation (3).


(2) $$d_{\mathrm{avg}\;}=\frac{d_{\mathrm{Wrist}-\mathrm{MCP}\;}+d_{MCP-PIP}+d_{PIP-DIP}}{3}$$

(3) $$DepthScaling=\left(\frac{d_{\mathrm{avg}}-d_{MCP-PIP}}{d_{\mathrm{avg}}}\right)\left(d_{max}-d_{min}\right)$$

Next, we assign depth values to each joint to create pseudo-3D coordinates based on the *DepthScaling*. Specifically, the MCP, PIP, and DIP joints receive progressively larger depths with Equations (4)–(6). These depth values, combined with the 2D joint positions and camera intrinsic parameters, are used to reconstruct the pseudo-3D coordinate (X,Y,Z) from each 2D joint position (u,v) through the pinhole camera model using Equation (7).


(4) $$Z_{MCP}=0.8\mathrm{DepthScaling}$$

(5) $$Z_{PIP}=0.9\mathrm{DepthScaling}$$

(6) $$Z_{DIP}=\mathrm{DepthScaling}$$

(7) $$X=\left(u-c_{x}\right)\frac{Z}{f_{x}},Y=\left(v-c_{y}\right)\frac{Z}{f_{y}}$$

Where Z is the assigned depth for that joint (e.g., $Z_{MCP},\;Z_{PIP},\;Z_{DIP}$).

### Ray casting

**Figure 2.**
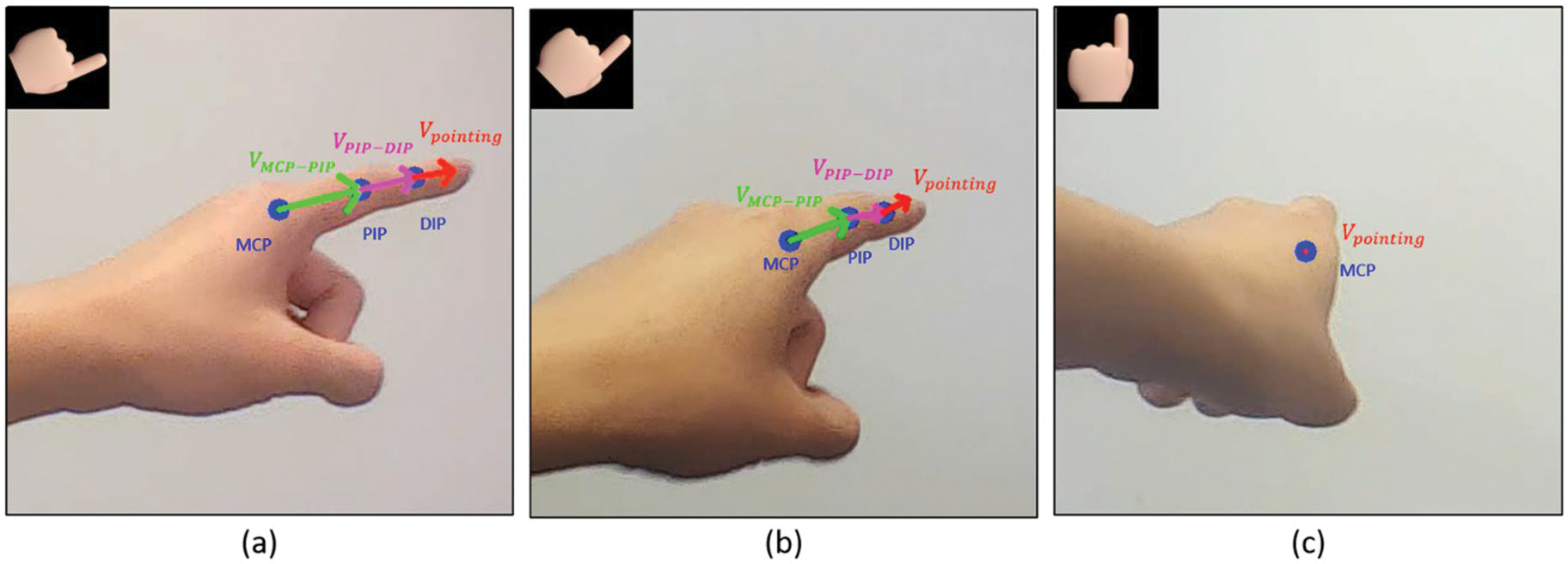
Visualisation of the pointing vector computation based on index finger landmarks. The key joints are marked in blue, including the MCP, PIP, and DIP joints. The segment vectors are colour-coded: $v_{\mathrm{MCP-PIP}}$ (green) represents the vector from MCP to PIP, $v_{\mathrm{PIP-DIP}}$ (pink) represents the vector from PIP to DIP, and the pointing vector $v_{\mathrm{pointing}}$ (red) is computed as a weighted combination of the $v_{MCP-PIP}$ and $v_{PIP-DIP}$. The top-right corner of each image presents the top view of the hand, illustrating the transition of the pointing direction from the right side to forward in (a) through (c). In (b), the red vector is shorter than in (a), indicating a deeper pointing direction. In (c), the MCP replaces the DIP as the ray’s origin due to complete occlusion of the index finger where the ray direction is set straight forward.

We compute direction vectors for each finger segment by subtracting the 3D positions of consecutive joints, $v_{MCP-PIP}$ and $v_{PIP-MCP}$. These vectors represent the orientations of the finger segments in 3D space. To determine the overall pointing direction, we combine these segment vectors using weighted coefficients that reflect the contribution of each segment to the pointing gesture. For instance, the vector from the MCP to the PIP joint may be given a higher weight, as it demonstrates more consistent detection accuracy by the hand pose estimation model than PIP-DIP (based on our observations). The combined vector is then normalised to obtain a unit vector representing the ray casting direction with Equation (8). When the back of the hand blocks the view of the camera capturing the index finger, and the index finger is almost obscured, the system will utilise the MCP as the ray casting origin and set the direction directly forward (see Figure 2).


(8) $$v_{\mathrm{pointing}}=\frac{\omega_{1}v_{MCP-PIP}+\omega_{2}v_{PIP-DIP}}{\left\|\omega_{1}v_{MCP-PIP}+\omega_{2}v_{PIP-DIP}\right\|}$$

Where $\omega_{1}=0.7,\;\omega_{2}=0.3$ are the weighting coefficients, and $v_{\mathrm{pointing}}$ is the normalised pointing direction vector.

### Confidence score calculation

**Figure 3.**
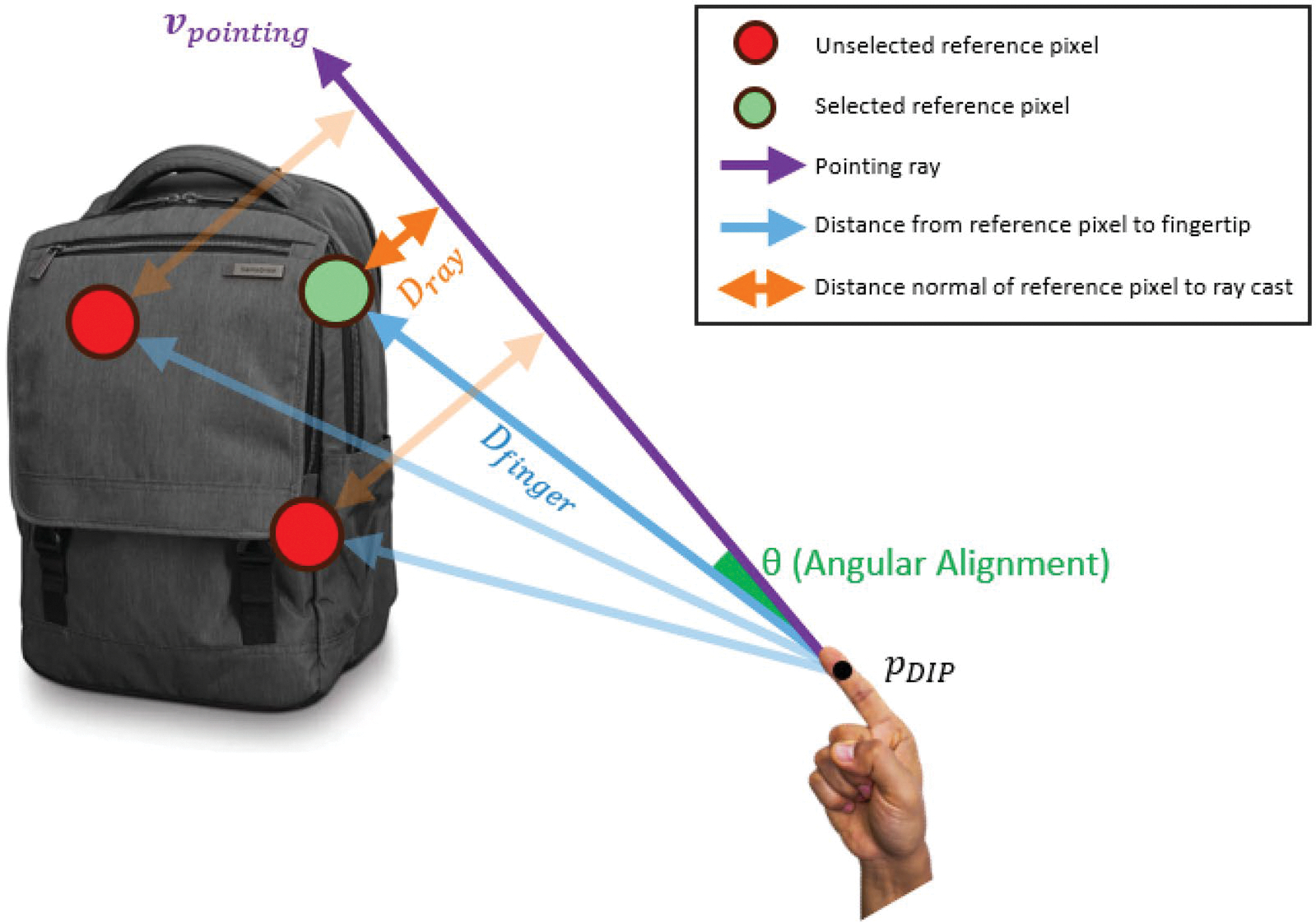
Comparative illustration of the confidence score evaluation for pointing ray-based object selection. The pointing ray ($v_{\mathrm{pointing}}$) originates at the DIP joint ($p_{DIP}$) and extends outward. For simplification, only three reference pixels are illustrated to represent the pixel of the backpack. The green circle represents the reference pixel with the highest confidence score in this case. The confidence score is calculated based on the following factors: Distance normal of reference pixel to ray cast $\left(D_{\mathrm{ray}}\right)$: The distance of object pixels to the ray is compared. The reference pixel, with a shorter distance, receives a higher confidence score for this aspect than the rest pixels. Angular Alignment (θ): The green pixel with a smaller deviation angle, has a higher alignment confidence score than the two red pixels. A smaller angle indicates closer alignment with the pointing ray. Distance from reference pixel to fingertip ($D_{\mathrm{finger}}$): This factor adjusts the confidence score based on the distance between the reference pixel and the fingertip. While the confidence score decreases as the distance increases, the relationship is not strictly linear. This ensures that reference pixels at greater distances could still be viable candidates if their alignment and proximity scores are strong, while closer pixels naturally receive higher confidence due to their proximity.

The confidence score is designed to evaluate the likelihood that a given object is being pointed at by the user in three dimensions. This score integrates three factors: distance normal of reference pixel to ray cast, angular alignment, and distance from reference pixel to fingertip. Figure 3 illustrates the concept used for the confidence score calculation.

### Distance normal of reference pixel to ray cast.

This factor measures how close each object’s pixels are to the ray cast from the user’s pointing direction. The ray originates from the user’s index finger $p_{DIP}$ and follows the direction $v_{\mathrm{pointing}}$. For each given 3D point $p_{\mathrm{obj}}\left(X_{\mathrm{obj}},Y_{\mathrm{obj}},Z_{\mathrm{obj}}\right)$, the distance $D_{\mathrm{ray}}$ from $p_{\mathrm{obj}}$ to the ray origin $p_{DIP}\left(X_{DIP},Y_{DIP},Z_{DIP}\right)$ is computed using the cross product in Equation (9),

(9) $$D_{\mathrm{ray}}=\frac{\left\|\left(p_{\mathrm{obj}}-p_{DIP}\right)\times v_{\mathrm{pointing}}\right\|}{\left\|v_{\mathrm{pointing}}\right\|}$$

To normalise this component, we define the ray distance component $C_{\mathrm{ray}}$ as in Equation (10),

(10) $$C_{\mathrm{ray}}=\frac{1}{D_{\mathrm{ray}}+1}$$

### Angular alignment.

This factor assesses the angular alignment between the ray direction and vectors pointing from the ray origin to each pixel on the object. For each point $p_{\mathrm{obj}}$, we compute the vector $q=p_{\mathrm{obj}}-p_{DIP}$ and normalise it to obtain $q_{\mathrm{norm}}=\frac{q}{\left\|q\right\|}$. The cosine of the angle θ between $q_{\mathrm{norm}}$ and the ray direction $v_{\mathrm{pointing}}$ is calculated using the dot product in Equation (11),

(11) $$cos\theta =q_{\mathrm{norm}}\cdot v_{\mathrm{pointing}}$$

This cosine value represents the alignment, with values closer to 1 indicating better alignment.

Distance from reference pixel to fingertip: This component accounts for the distance between the object’s pixel-by-pixel and the ray origin. This component slightly adjusts the system’s sensitivity to deviations in angle and proximity by the object’s distance. The distance from each point to the ray origin is $D_{\mathrm{finger}}=\|q\|$. We define the finger distance component $C_{\mathrm{finger}}$ as the normalised distance with Equation (12),

(12) $$C_{\mathrm{finger}}=\frac{D_{\mathrm{finger}}}{D_{\mathrm{finger}}^{\max}}$$

where $D_{\mathrm{finger}}^{\max}$ is the maximum considered distance, here we set it as 20 metres. The distance scaling factor $S_{\mathrm{distance}}$ is then computed using Equation (13),

(13) $$S_{\mathrm{distance}}=\frac{1}{1\;+\;\alpha \;\cdot \;C_{\mathrm{finger}}}$$

where α is a scaling constant that adjusts the influence of the distance. Here the α is 0.5.

The final confidence score C for each pixel is calculated by combining these components using a weighted sum,

(14) $$C=S_{\mathrm{distance}}\left[\beta \cdot C_{\mathrm{ray}}+(1-\beta )\cdot cos\theta \right]$$

where β=0.3 is a weighting factor that balances the influence of the distance normal to ray cast and the angular alignment.

**Figure 4.**
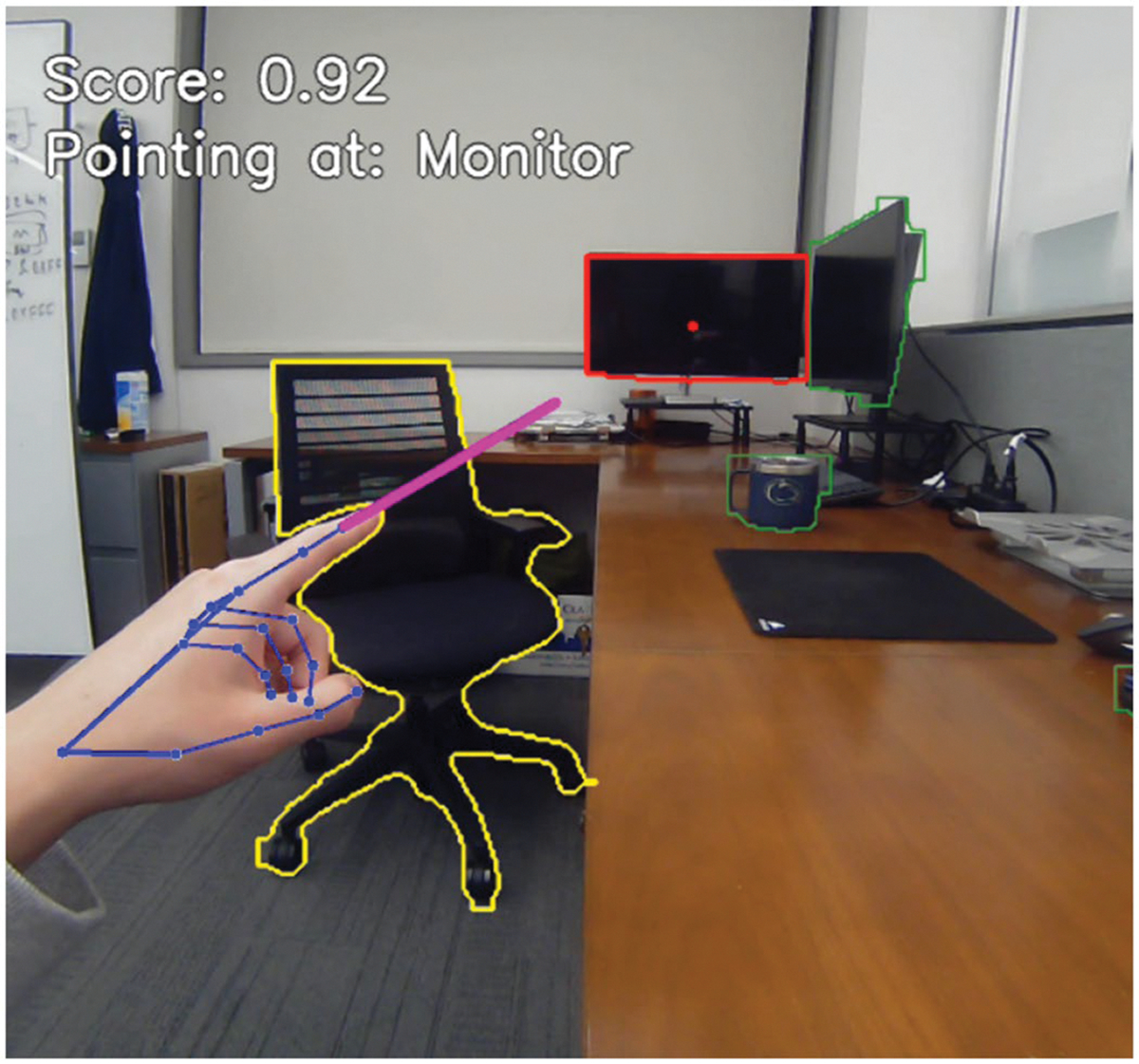
Visualisation of the system’s functionality, showcasing hand pose estimation, object segmentation, and ray-casting for object identification. The user points at a monitor with the left hand, and the system assigns a confidence score of 0.92. The chair (yellow outline) represents an object that would have been selected as the pointed object in the previous version of the system, where fingertip position alone determined selection. The selected monitor (red outline) is the object identified by the current system, while objects (green outlines) are detected but not selected. The purple line visualises the calculated ray direction in 3D space projected back into the 2D image. A red dot on the monitor represents the selected point, determined based on the highest confidence score. Blue dots and connecting lines illustrate the estimated hand landmarks and skeletal structure, as detected by the hand pose estimation model.

The overall confidence score for an object is determined by the highest confidence score among all its pixels. After computing confidence scores for all objects in the frame, the system selects the object with the highest score, provided it exceeds a predefined threshold of 0.7. This threshold value accounts for inaccuracies in the depth estimation module and ray-casting module thus ensuring that only the most likely target object is considered, reducing ambiguity and enhancing reliability in object selection based on the user’s pointing gesture. The threshold also serves to prevent the selection of objects when the user is pointing at empty space, further improving the accuracy and relevance of the system’s output. see Figure 4 for a visualisation.

### Audio feedback

To generate the speech output, our system utilises the VITS (Variational Inference with adversarial learning for end-to-end Text-to-Speech) model, a non-autoregressive architecture designed to directly predict speech waveforms from input text [40]. This model allows for rapid and efficient speech synthesis, enabling real-time auditory feedback. The output module is programmed to announce the currently selected object every three seconds and provide the distance information rounded to the nearest 0.5-metre increment. Additionally, when the system detects a new pointed object, the system will immediately announce the name of the object regardless of the three-second interval, ensuring timely and relevant information is conveyed to the user as they explore the scene.

### Experiment

We designed a technical validation to assess the object identification accuracy of the system across various horizontal positions at distances ranging from 1 to 5 m within an indoor environment under natural light conditions. The validation aimed to simulate real-world scenarios by incorporating various common objects such as phones, bottles, laptops, backpacks, chairs, and suitcases. Except for the chair, all objects were placed on a table at a height of 1.5 m to maintain consistent elevation from the floor during the experiment preventing variations in vertical positioning that could arise at greater distances.

**Figure 5.**
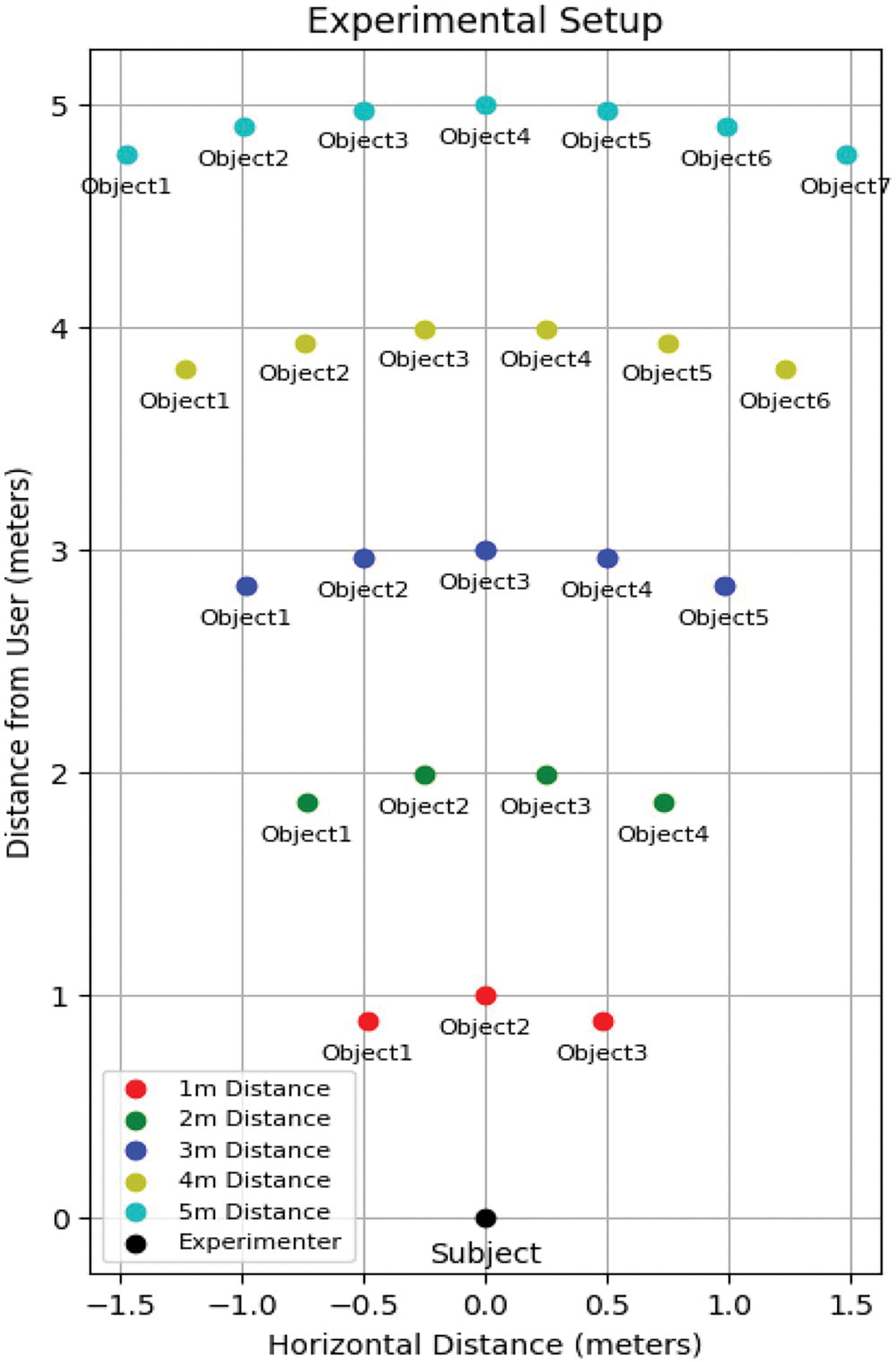
Experimental setup for validation testing, with objects positioned at increasing distances (1 to 5 metres) from the subject at the origin (black marker). Objects are evenly spaced at each distance to ensure consistent conditions for evaluating system performance.

At the 1 m distance, testing involved three objects arranged in a curve, ensuring the distance to the subject consistent; objects were spaced with equal distances (inter-object spacing). For each subsequent metre, an additional object was added to the array, maintaining uniform spacing between all objects (See Figure 5). Small objects, such as a phone, bottle, and bowl, were used for shorter distances (1–2 m), while larger objects, including a backpack, suitcase, and chair, were selected for greater distances so that the object’s visual size in the image maintained greater consistency. This systematic arrangement progressively increased the complexity of the environment, while ensuring controlled object density and spacing, facilitating a robust evaluation of the system’s performance.

In the validation tests, subjects wore the backpack with the integrated system and had a laser pointer mounted on the index finger of his dominant hand. The laser pointer provided a visual reference by projecting a laser dot onto the object being pointed at. This ensured the subject could consistently verify and maintain alignment with the correct object throughout the experiment, offering a reliable ground-truth reference for system evaluation. The camera was mounted on the left strap of the backpack, and the subject pointed at an object with the right hand. The order in which the object was pointed was determined by a pre-generated random sequence representing the object left to right indices. The subject pointed at each object, with confirmation provided by the laser pointer indicator, for approximately 60 s to ensure that sufficient data were collected. This process was repeated until all the objects had been pointed at.

The validation test focused on four key performance metrics:

1. Hand Pose Estimation Classification: Determined by the number of frames where the hand pose was successfully estimated out of the total frames captured. A hand was considered successfully detected if the estimated key points formed a plausible skeletal structure, which was visually inspected for alignment with typical hand configurations during pointing gestures.
2. Object Recognition Accuracy (YOLO Recognition Accuracy): Calculated based on the number of frames where the YOLO algorithm correctly detected and classified the laser-verified pointed object.
3. Pointing-based Object Selection Accuracy: Assessed by the number of frames where the system correctly determined the object being pointed at. This also includes frames where object classification failed but the object was detected as the pointed target correctly.
4. Object Distance Detection: Evaluated by analysing the system’s estimated distances for the pointed objects across all frames. Statistical metrics such as the average, maximum, minimum, median, and variance of the estimated distances are used to quantify the system’s consistency and stability in determining the spatial positions of the objects.

## Results

### Hand pose estimation classification

The hand pose estimation classification result demonstrated consistent performance across all the tests, with the detected hand keypoints aligning with plausible skeletal configurations during pointing gestures. Across all evaluated distances, the system achieved a 99% accuracy rate based on manual verification. This consistency in hand pose estimation was critical for the system’s overall performance, enabling reliable ray-casting and object selection across varying distances.

### YOLO object recognition accuracy

Object recognition accuracy showed a clear decline as the distance increased. At 1 m, YOLO maintained high recognition accuracy, averaging above 95% for most objects. However, at 5 m, recognition accuracy dropped strongly for certain objects, such as backpacks (3.13%). These results demonstrate the limitations of YOLO’s performance at greater distances, probably due to reduced image size and loss of image detail/clarity. Nonetheless, some objects, such as the laptop and chair, maintained high accuracy even at 5 metres, reaching 95.83% and 99.14%, respectively.

### Pointing-based object selection accuracy

**Figure 6.**
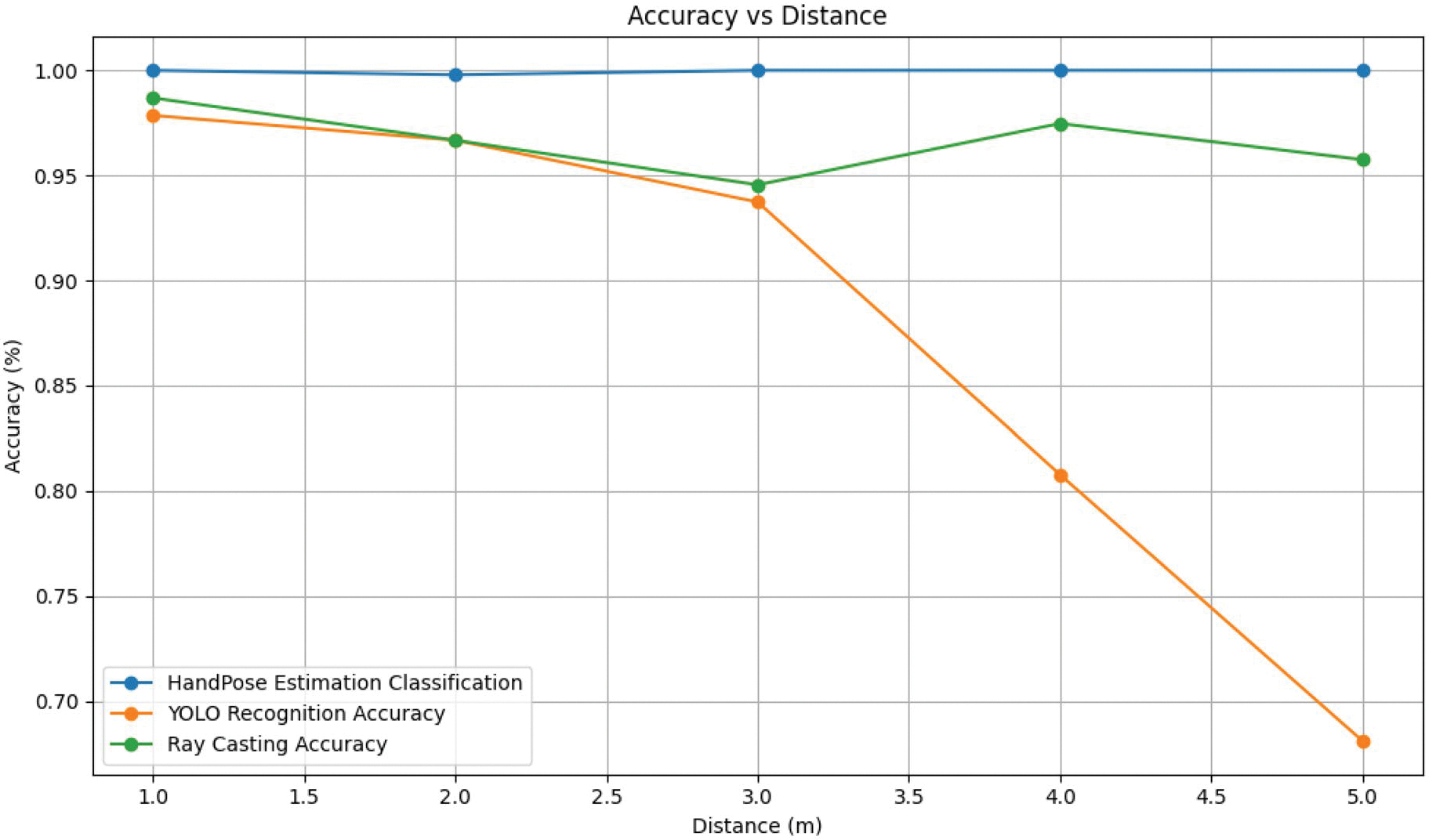
Accuracy of System Components Across Distances. The graph illustrates the performance of three key system metrics—Hand Pose Estimation Classification, YOLO Recognition Accuracy, and Pointing Object Detection Accuracy—measured at distances ranging from 1 to 5 m. Hand Pose Estimation consistently achieves near 100% correct classification. Pointing Object Detection Accuracy remains stable until slight deviations occur at larger distances. YOLO recognition accuracy, while initially comparable to other components, strongly decreases at 3 m, indicating the worse performance of object detection at greater distances.

The pointing object detection module exhibited strong stability across all distances, consistently achieving high accuracy while YOLO object recognition accuracy declined. The pointing-based object selection accuracy for the same object was 96.90% among the total frames. This resilience underscores the effectiveness of the ray casting and confidence score approaches in accurate detection of the object being pointed at. The above three metrics results are shown in Table 1 and plotted in Figure 6.

### Distance of the pointed object

**Figure 7.**
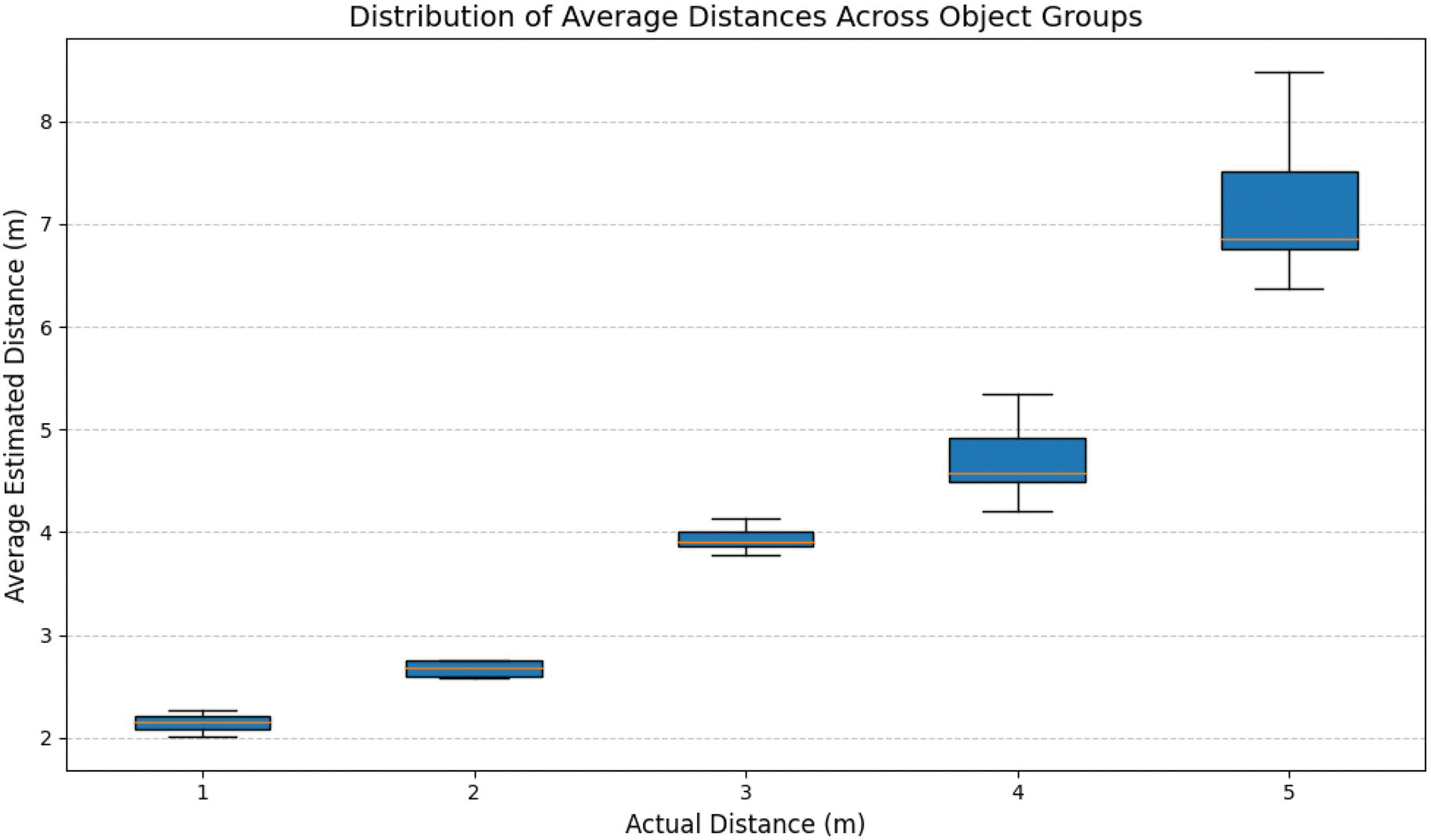
Box plot showing the distribution of average distances across object groups for each distance from the user. The x-axis represents the distance categories (1 m to 5 m), while the y-axis indicates the average estimated distances in metres by the system. The boxes illustrate the interquartile range (IQR) of average distances, with whiskers extending to the minimum and maximum values within the range. This plot indicates the increasing spread and central tendency of average distances as the distance from the user grows.

Distance estimation for pointed objects was evaluated using average, maximum, minimum, median, and variance metrics across distances from 1 m to 5 m (Table 2). Figure 7 shows the distribution of estimated distances for each actual distance. At 1 m and 2 m, variance was minimal (below 0.04), with medians closely matching averages (e.g., a phone was estimated at 2.01 m on average with a median of 1.96 m). At 3 m, estimates showed moderate variance with averages and medians remaining in close agreement. At 4 m and 5 m, variance increased, for instance, a backpack at 5 m exhibited a variance of 2.25, though median values still clustered around the average estimates. Overall, the system overestimated distances by 1.21 m.

## Discussion

### Analysis of the results

The proposed system demonstrates promising results for enabling the accurate detection and selection of objects being pointed at by the user within a 5-metre range. By integrating depth estimation, hand pose recognition, and object segmentation, the system effectively identifies the object of interest and provides spatial information through audio feedback. The system was technically validated across four components: hand pose estimation classification, the hand was detected near 100% across all tests; YOLO object recognition demonstrated reliable performance within 3 metres, maintaining over 90% accuracy, but its performance declined beyond this range. Pointing object detection accuracy remained robust up to 5 metres. Distance estimation exhibited a consistent bias, with predicted object distances systematically overestimated by approximately 1 m across all tested ranges. This performance from the validation test indicates the potential of the system as a valuable assistive tool for pBLV.

### System requirements

The system integrates multiple forms of AI using both serial and parallel processing streams. By adopting the combinatorial AI framework, the system ultimately achieves around 3–5 frames per second during operation. Improving the performance of each underlying process or module, alongside more seamless integration should improve this metric and bring the device further in line with real-time processing for the user.

### Computer vision for object detection

Overall, the system demonstrates solid performance throughout the validation. However, the object detection module exhibits certain limitations, as the distance between the user and the objects increases. Specifically, the accuracy of object recognition and segmentation decreases noticeably at 3 and 4 m, with a substantial decrease in performance at 5 m. This decline may be attributed to the reduced image size of objects within the camera frame at greater distances, which results in fewer pixels representing each object. The diminished pixel representation adversely affects the detection model’s ability to accurately identify and segment objects, as smaller objects are more susceptible to noise and less distinguishable from the background [41]. Additionally, factors such as lighting conditions, occlusions, and perspective distortion become more pronounced at longer distances, further challenging the object detection algorithm’s effectiveness. These limitations highlight the need for advanced detection models capable of maintaining high accuracy across varying distances and for techniques that enhance object representation in the image, such as super-resolution [42,43] or adaptive scaling [44]. Specifically, higher resolution sub-regions of image frames could be dynamically processed for the regions being pointed at, improving the system’s ability to discern finer details and maintain robustness in challenging scenarios.

To mitigate the adverse effects of low-light conditions on object detection, various image enhancement techniques can be integrated into our system. Recent advancements in low-light image enhancement techniques such as greyscale transformation [45], retinex methods [46] and machine learning methods [47] can be adopted to further improve the reliability of the system when the user is within a low-illuminance environment.

Currently, the system is limited to detecting and recognising 80 object classes from the COCO dataset due to the constraints of the YOLOv11 model’s pre-trained configuration. While this covers many commonly encountered objects, it may not include items that are specific to certain environments or tailored to the unique needs of pBLV, such as mobility aids, household appliances, or specialised tools. Expanding the range of detectable object classes by incorporating additional training datasets or fine-tuning the model with custom annotations could significantly improve the system’s relevance, versatility, and adaptability [48,49]. This enhancement would enable the system to cater to a broader range of use cases and provide more meaningful and context-aware feedback to users, further increasing its utility and impact.

Within the segmentation pipeline, the YOLO segmentation requires mobileSAM in order to filter the segmentation results, which increases the workload of our system. Addressing this issue would involve optimising the segmentation pipeline, such as developing a more efficient integrated model that combines object detection and high-precision segmentation without the need for integrating independent models. This optimisation could enhance the system’s responsiveness and make it more suitable for practical, real-world applications where timely feedback is crucial.

### Computer vision for depth estimation

The system achieves near-real-time performance, which indicates an area for further optimisation, particularly in dynamic environments where faster feedback is essential to ensure seamless user interaction. The primary bottleneck in the system’s performance is the depth estimation module, specifically the Depth Anything model. Depth estimation is computationally intensive, especially when working with high-resolution frames and performing frame-by-frame analysis in real time on the limited performance hardware. Optimising this module, through either incorporating distance-estimating sensors [50,51], or exploring lightweight distance estimation alternatives could significantly enhance processing speed. Additional strategies include leveraging hardware acceleration, adopting parallel processing techniques to distribute computational loads, or utilising a higher-performance edge device capable of handling intensive computation with reduced latency [52]. Addressing these aspects would improve overall system responsiveness, ensuring it meets the demands of real-world, dynamic scenarios.

In addition, we have variability in the performance of the depth estimation module across different types of predictions during the development. Specifically, the model exhibits poorer performance when estimating the depth of human hands compared to its accuracy in predicting environmental features such as objects. This disparity likely arises from the composition of the pretrained model’s training dataset, which appears to lack sufficient examples of human body parts, particularly hands from a first-person perspective. The lack of diverse and representative training data likely hinders the model’s ability to generalise to such scenarios. Depth estimation for human hands is also challenging due to their complex and highly articulated structure, as well as frequent occlusions and variations in pose [53]. Additionally, the proximity of the hand to the camera in the current system setup, designed for capturing the front view from the user, may further contribute to the difficulty, as objects closer to the camera may fall outside the optimal focus range, potentially leading to greater estimation errors due to defocus blur and limited depth of field.

The distance results also suggest a systematic bias, as depth predictions consistently overestimate ground truth distances by approximately 1 m. This bias may stem from the distortion introduced by the fisheye camera’s wide field of view, which can affect depth estimation by exaggerating the apparent distance of objects. Additionally, inaccuracies in the depth estimation model, potentially influenced by dataset biases or camera calibration limitations, may further contribute to this overestimation. Future work will explore potential methods to address this limitation, including calibrating the depth estimation pipeline, incorporating additional training data tailored to the system’s use case, or implementing post-processing corrections to mitigate the observed bias.

### Proprioceptive integration for enhanced spatial mapping and action guidance

Building on our innate proprioceptive sense provides a natural egocentric framework for spatial understanding and action guidance. By harnessing inherent body awareness, the system leverages the user’s pointing direction to directly register an object’s azimuth and elevation angles relative to the users’ body. This direct mapping bypasses the need for additional auditory encoding, such as using pitch for verticality or spatialised sound for laterality, thereby reducing cognitive load and enabling users to construct accurate mental maps of their surroundings. Recent studies have demonstrated that augmenting sensory substitution with proprioceptive cues can improve spatial cognition and facilitate more intuitive action planning. For example, research by Abboud et al. [2] on the EyeMusic SSD and subsequent work by Maidenbaum et al. [54] show that when users’ own motor signals are integrated into the feedback loop, perceptual learning is enhanced and object relationships become clearer. This embodiment of perception, wherein the body itself serves as the reference point, not only heightens environmental awareness but also guides user actions more effectively, aligning sensory feedback with natural motor behaviour.

### Future work

Future work will focus on enhancing the system’s robustness and usability in real-world scenarios by designing experiments with greater ecological validity. This will include conducting studies with pBLV to gather insights into practical challenges, user behaviour, and system interaction during basic and instrumental activities of daily living [55]. This study presents an approach that allows users to obtain supplementary environmental information through pointing gestures. Crucially, this is done without requiring hardware that is either on the hands, or hardware that needs to be physically interacted with. This hands-free design makes the system particularly suitable for tasks where both hands are occupied. For instance, activities such as cooking or washing dishes can benefit from intuitive hand gestures and audio feedback, enabling users to access relevant information without disrupting their workflow. User studies focused on these ecologically valid tasks will help inform specific refinements to the system’s operation and interface to improve the system’s intuitiveness and practicality. Additionally, optimising lightweight models for faster inference and integrating advanced occlusion-handling algorithms will further enhance the system’s responsiveness.

A critical consideration is ensuring the system’s compatibility with common mobility aids, such as white canes and guide dogs. Orientation and mobility (O&M) training [56] typically advocates for keeping one hand free. While many alternative forms of assistance may occupy the users’ hands (e.g., smartphone Apps, electronic travel aid hardware), our system allows users to keep their hands free. This allows users to maintain readiness to brace against falls, free to hold additional objects (e.g., shopping bags) or to more easily interact with the environment (e.g., open doors). Future studies should explore how these forms of assistive technologies interact with primary mobility aids, or during specific tasks, and whether a broader range of gesture interactions during these moments can enhance compatibility and flexibility [57]. It should also be investigated as to whether the use of mid-air gestures can inadvertently interfere with established O&M safety practices.

The social acceptability of gestural interactions in public contexts is also essential to consider. Overt gestures might unintentionally draw attention, cause social discomfort by being misunderstood, or be considered intrusive in daily life. To enhance public acceptance and usability, future research should evaluate the use of discreet and context-sensitive interaction methods, including subtle gestures, brief verbal commands, camera-based object selection mechanisms, or the use of contextually-aware AI to intuit objects of interest to the user.

Future research will evaluate the current system’s feasibility and form factor [58], including when applied to alternative form factors such as on smart glasses or head-mounted alternatives and other approaches (e.g., cane-mounted, handheld). Such comparative analyses will address form factor trade-offs, user comfort, practicality during household tasks, and overall usability. While this system could utilise head-mounted cameras, there are some distinct advantages of body-mounted designs. In particular, they can provide more stable camera views that better avoid deterioration in image quality, are more likely to be aligned with users’ forward motion than head direction, avoid requiring or encouraging head movements that may run counter to O&M best practices (e.g., such as using head movements to better localise audible objects), or increase user discomfort through added weight or requiring specific head movements (e.g., looking down to view your hands). Future testing could compare head-mounted and body-based approaches during daily tasks to establish key advantages and pitfalls with each form factor.

Other future improvements may relate to improving how context-aware the system is when dealing with different environments or user intentions. In order to expand the system’s operational modes to support more flexible and context-aware interactions, the system could identify clusters of objects within the vicinity of the pointing direction, rather than isolating a single object. This functionality would be particularly useful for grouping related items, such as tools on a workbench or ingredients on a countertop. Additionally, the system could be adapted to recognise object pairs or combinations, enabling tasks such as matching shoes, identifying complementary items, or prioritise specific objects most relevant to the specific task being undertaken (for instance, prioritising feedback for the ingredients used in the current recipe). By incorporating these advanced interaction modes, the system could better address diverse user needs and further enhance its applicability in real-world scenarios.

## Conclusion

This paper introduces a novel assistive technology system that advances sensory substitution by integrating hand position sense (proprioception) with AI-driven mechanisms, bridging gesture recognition with spatial cognition for pBLV. By blending depth estimation, hand pose recognition, and object segmentation with a single monocular camera, the system achieves reliable and intuitive object identification through natural pointing. Validation testing demonstrates the system’s robustness in accurately identifying objects within a 5-metre range across a wide field of operation. The incorporation of a ray-casting mechanism and confidence-based object selection scoring ensures precise object targeting, focusing the visual analytics offered through computer vision advances.

## Figures and Tables

**Table 1.** Validation results across distances of 1–5 m.

Distance (m)	Objects (From Left to Right)	Total frames	HandPose estimation classification	YOLO recognition accuracy	Pointing-based object selection Accuracy

1	Phone	127	100.00%	95.28%	96.06%
	Bottle	118	100.00%	100.00%	100.00%
	Bowl	117	100.00%	98.29%	100.00%
2	Laptop	129	100.00%	97.67%	97.67%
	Backpack	131	100.00%	92.37%	92.37%
	Keyboard	119	99.16%	96.64%	96.64%
	Bottle	111	100.00%	100.00%	100.00%
3	Laptop	123	100.00%	91.06%	94.31%
	Backpack	119	100.00%	98.32%	99.16%
	Handbag	93	100.00%	90.32%	90.32%
	Laptop	172	100.00%	93.60%	93.60%
	Backpack	108	100.00%	95.37%	95.37%
4	Backpack	99	100.00%	71.72%	94.95%
	Chair	117	100.00%	99.15%	99.15%
	Backpack	117	100.00%	97.44%	97.44%
	Suitcase	109	100.00%	22.02%	98.17%
	Backpack	129	100.00%	99.22%	100.00%
	Laptop	185	100.00%	95.14%	95.14%
5	Chair	105	100.00%	97.14%	97.14%
	Backpack	96	100.00%	3.13%	93.75%
	Backpack	85	100.00%	30.59%	96.47%
	Laptop	96	100.00%	95.83%	88.54%
	Backpack	129	100.00%	53.49%	96.90%
	Suitcase	119	100.00%	97.48%	98.32%
	Chair	116	100.00%	99.14%	99.14%

For each object (listed from left to right), the table shows the total number of frames processed, hand pose estimation classification accuracy (verified *via* skeletal configurations), YOLO recognition accuracy, and pointing-based object selection accuracy. While hand pose estimation is nearly perfect at all distances, YOLO accuracy declines at longer ranges, yet the pointing-based selection remains robust.

**Table 2.** Distance estimation results.

Distance (m)	Objects (From Left to Right)	Average	Maximum	Minimum	Median	Variance

1	Phone	2.01	2.47	1.67	1.96	0.03
	Bottle	2.26	2.89	1.67	2.25	0.02
	Bowl	2.15	2.77	1.65	2.13	0.04
2	Laptop	2.75	3.39	2.02	1.81	0.08
	Backpack	2.58	2.84	2.26	2.58	0.01
	Keyboard	2.6	2.82	2.24	2.61	0.01
	Bottle	2.75	3.18	2.41	2.74	0.02
3	Laptop	4.01	4.67	3.57	3.99	0.06
	Backpack	3.86	4.28	3.52	3.86	0.03
	Handbag	3.9	4.73	0.88	4.07	0.59
	Laptop	4.13	1.05	2.76	3.97	0.26
	Backpack	3.78	4.17	3.38	3.75	0.03
4	Backpack	4.54	5.32	3.81	4.57	0.06
	Chair	4.61	5.34	2.83	4.63	0.28
	Backpack	5.35	6.04	1.9	5.38	0.18
	Suitcase	4.21	5.5	1.27	4.7	1.31
	Backpack	4.48	4.89	4.23	4.44	0.03
	Laptop	5.02	5.39	4.56	5.04	0.02
5	Chair	7.36	8.33	6.32	7.32	0.24
	Backpack	8.48	9.58	1.67	8.74	1.77
	Backpack	6.72	8.06	1.5	6.7	0.43
	Laptop	6.79	7.71	6.32	6.71	0.11
	Backpack	6.37	7.88	1.05	6.71	2.25
	Suitcase	7.66	8.44	7.04	7.63	0.09
	Chair	6.85	8.03	6.21	6.86	0.04

This table reports the system’s distance estimation performance for objects placed at nominal distances ranging from 1 m to 5 m. For each object category (e.g., phone, bottle, laptop, backpack, etc.) arranged by distance (from left to right), the table reports the average, maximum, minimum, and median estimated distances along with the variance. The results indicate that at shorter distances (1–2 m), estimates are highly consistent (variance < 0.04 and medians nearly equal to averages), while at longer distances (3–5 m) the variability increases, indicating greater uncertainty in depth estimation. Overall, the system exhibits a mean overestimation bias of approximately 1.21 m.

## IMPLICATIONS FOR REHABILITATION

- Enhanced Spatial Awareness: By leveraging hand proprioception and accurate 3D ray-casting, the system is expected to help users develop more robust mental maps of the environment by building off an egocentric representation of space. Pending further validation, this potential improvement in spatial awareness may enhance visual rehabilitation for orientation, mobility, and awareness of key objects in the environment.
- Reduced Cognitive Load: This gesture-based method allows the user to focus the AI feedback on describing specific elements of a scene. This potentially allows the user to focus on relevant information to them, while minimising extraneous information. This could reduce the cognitive burden associated with systems that provide too much irrelevant information that the user must then parse through.
- Increased Independence in Daily Activities: The hands-free design of the technology should allow the user to be able to focus on interactions with the environment, rather than on manipulating hardware. This should make users more efficient, in being able to call on additional information through brief mid-air gestures, even in the middle of completing daily tasks.
- Integration with Personalised Rehabilitation Programs: The adaptive and context-aware nature of the system makes it a promising candidate for inclusion in comprehensive rehabilitation protocols. Further optimisation and experimental testing of the system will aim to tailor sensory feedback specifically to support user-centric learning and functional improvements while ensuring privacy safeguards and confirming efficacy through testing with persons who are visually impaired.
